# Fluoroscopic views for safe insertion of lag screws into the posterior column of the acetabulum

**DOI:** 10.1186/1471-2474-15-303

**Published:** 2014-09-15

**Authors:** Wei Chen, Zekun Zhang, Yang Lu, Jia Li, Yingze Zhang, Yong Shen

**Affiliations:** Department of Orthopaedic Surgery, Third Hospital of Hebei Medical University, Shijiazhuang, Hebei 050051 P.R. China; Department of Radiology, Third Hospital of Hebei Medical University, Shijiazhuang, Hebei 050051 P.R. China

**Keywords:** Acetabular fracture, Posterior column, Percutaneous fixation, Fluoroscopic view

## Abstract

**Background:**

Percutaneous lag screw fixation is an alternative treatment for non-displaced or minimally displaced posterior column fractures. This study aims to explore new fluoroscopic views of the acetabulum for safe percutaneous insertion of posterior column lag screws.

**Methods:**

Axial computed tomography (CT) scans were taken of sixteen embalmed adult cadavers. The axial CT images at the level of the middle height of the acetabulum were selected. The angle (angle α) between the posterior cortex of the posterior column (PCPC) and the line intersecting the axial plane and the coronal plane, and the angle (angle β) between the medial wall and the line intersecting the axial plane and the sagittal plane were identified and measured. Tangential views of the PCPC and medial wall were obtained by referencing the measured angles. A lag screw was inserted into the posterior columns of the sixteen pelvic specimens under fluoroscopic guidance using an iliac oblique view and the two tangential views. CT scans were performed to evaluate the lag screw position. Axial CT images of 52 volunteers were obtained and the angles α and β were measured following the same methods used for the cadaveric specimens.

**Results:**

The angles α and β for the specimens were 29.3±2.8.1 and 8.1±1.4 degrees, respectively. On the tangential view of the PCPC, the posterior cortex appears as a nearly straight line between the lesser and greater sciatic notches. On the tangential view of the medial wall, the medial wall appears as a distinct straight line. Using these radiographic images, the lag screws were inserted into the posterior columns of bony pelvic specimens. Screw placement was confirmed by CT, and found to be fully intraosseous in all cases without any cortical breaches. The angles α and β were 30.4±4.1 and 9.2±1.9 degrees for male volunteers and 28.5±3.7 and 7.7±1.8 degrees for female volunteers, significant difference in these angles between cadaveric specimens and human volunteers.

**Conclusion:**

The tangential views of both the PCPC and medial wall can be obtained following the aforementioned methods The oblique iliac view and the two tangential views enable safe insertion of posterior column lag screws.

**Electronic supplementary material:**

The online version of this article (doi:10.1186/1471-2474-15-303) contains supplementary material, which is available to authorized users.

## Background

Fractures of the posterior column are a common subtype of acetabular fractures. Percutaneous lag screw fixation is an alternative approach for non-displaced or minimally displaced (<2 mm) posterior column fractures in patients with severe soft tissue injury, burns, and an increased risk for major surgery[1–6]. Percutaneous lag screws can also be used to fix a well-aligned acetabular fracture non-union[7] or act as an adjunct to traditional open reduction and internal fixation[4, 8]. Starr et al. applied this technique in the treatment of displaced acetabular fractures after reduction was performed in a closed or limited open fashion[6].

Percutaneous fixation can provide enough stability for early mobilisation[4, 6, 9], which is very important to avoid complications associated with prolonged bed rest[1]. Additionally, the dense scar tissue, contractures and problematic hardware commonly encountered after failed open operation of acetabular fractures can be avoided with the percutaneous technique[10]. However, this procedure is technically demanding and limited by narrow bony corridors[11]. There are concerns about violating the lateral wall of the acetabulum, resulting in articular penetration during lag screw fixation[12]. The lag screw may also protrude through the medial wall and jeopardise intrapelvic organs[13]. The sciatic nerve lies close to the posterior wall and is susceptible to injury in acetabular fractures involving the posterior wall and column[14]. The inferior gluteal neurovascular bundles also lie close to the posterior cortex of the posterior column (PCPC). The placement of lag screws into the posterior column may inadvertently penetrate the PCPC and damage the neurovascular structures[1]. Therefore, it is essential to improve the accuracy of lag screw insertion into the posterior column to reduce the risk of iatrogenic injury to the hip joint, adjacent neurovascular structures and intrapelvic organs[13, 15].

To guarantee safe percutaneous insertion of the lag screw into the posterior column, a conventional image intensifier is frequently used during the operation[13]. Multiple C-arm imaging at different angles is required for safe intraosseous placement of pelvic screws. The anteroposterior view of the pelvis and iliac oblique and obturator oblique views[16–22] are commonly employed to ensure that the guide wire does not penetrate the hip joint, medial wall or PCPC. However, misplacement of the posterior column lag screws still occurs in some cases. Consequently, it would be beneficial to explore new projections for intraoperative fluoroscopic guidance of percutaneous screw insertion. Therefore, the purposes of the present study were to 1) determine the optimal fluoroscopic angles for visualisation of the PCPC and medial wall of the acetabulum in cadaveric specimens, 2) confirm the effectiveness of these angles in ensuring intra-osseous positioning of retrograde percutaneous posterior column screws, and 3) evaluate whether fluoroscopic angles for visualisation of the posterior column in human subjects are similar to those identified in cadaveric specimens.

## Methods

**Figure 1:**
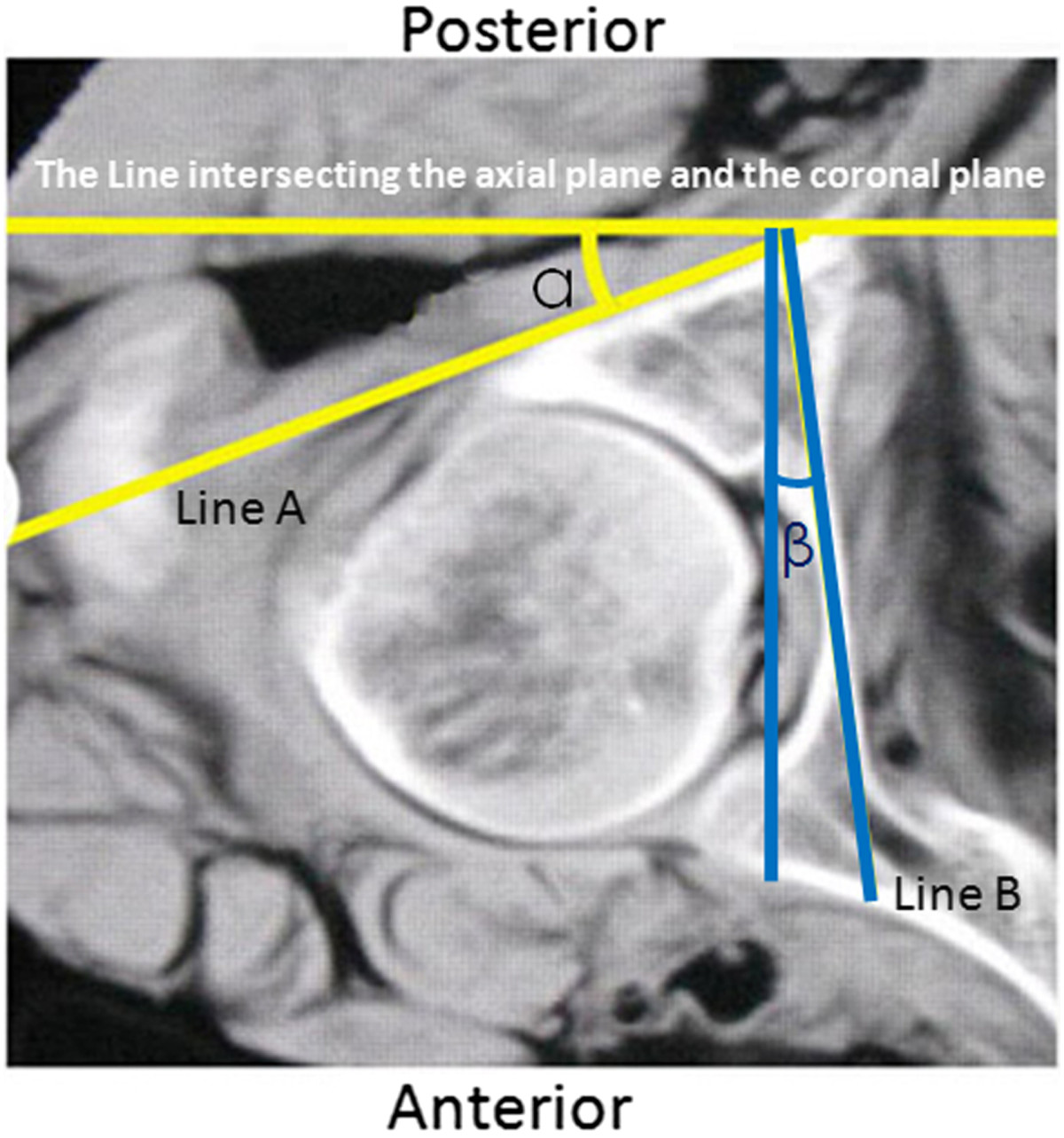
**On the axial computed tomography (CT) image at the middle height level of the acetabulum, Line A represents the posterior cortex of the posterior column.** The angle between Line A and the line intersecting the axial plane and the coronal plane is labelled angel α. Line B represents the medial wall of the acetabulum. The angle between Line B and the line intersecting the axial plane and the sagittal plane is labelled angle β.

**Figure 2:**
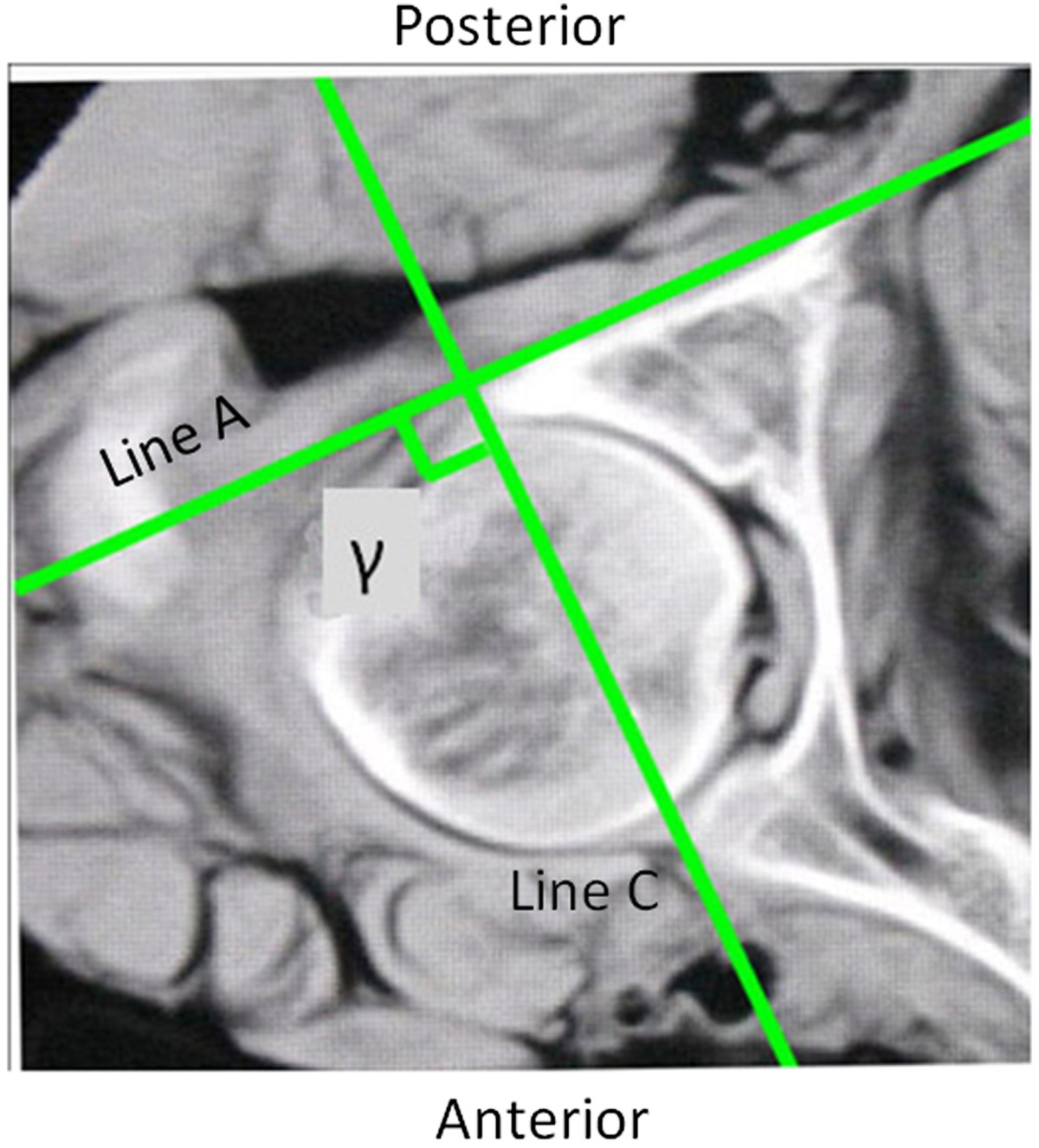
**On the axial CT images, Line C represents the line running through the anterior and posterior points of the lateral brim of the acetabulum.** The angle between Lines A and C is labelled angle γ.

**Figure 3:**
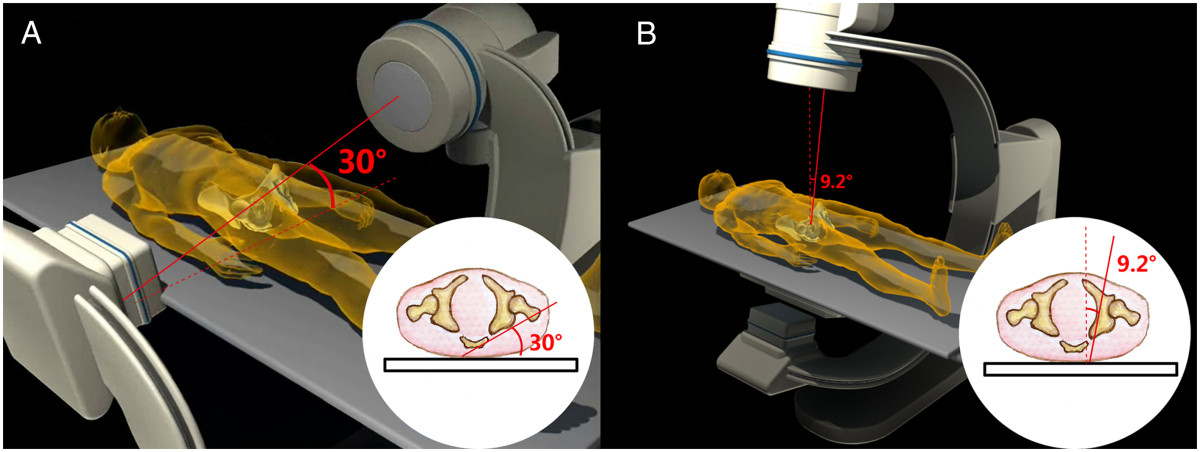
**The diagrams illustrate the position of the C-arm unit and the male patient to obtain the tangential views of the posterior cortex of the posterior column (A) and the tangential view of the medial wall of the acetabulum (B).**

Sixteen embalmed adult cadavers were obtained from the Department of Anatomy of Hebei Medical University (Shijiazhuang, China). The specimens were all males with an average age of 41 years (range, 25–70 years). The specimens were placed in the supine position on a radiolucent carbon fibre table. The longitudinal axis of the specimen was parallel to that of the table. CT scans were performed on all specimens using a commercially available Siemens spiral 64-slice multi-detector scanner (Siemens Medical, Nuremberg, Freistaat Bayern, Germany). The technical factors were 80–110 mAs, 120 kV, pitch 0.9 and an acquisition thickness of 0.75 mm. Axial images with a 2-mm slice thickness were created. The CT images of each specimen were reviewed and no bony deformity was noted. Based on a previous anatomical study that reported that the smallest axial cross-section of the posterior column is at the middle height level of the acetabulum[23], the axial CT image at the level of the middle height of the acetabulum was selected. The PCPC was identified on the selected CT image and labelled Line A (Figure 1). The line intersecting the axial plane and the coronal plane was marked, defined as the line tangential to the most posterior points of both acetabula. The angles between Line A and the line intersecting the axial plane and the coronal plane were measured using the measurement software MB ruler (Markus Bader, Iffezheim, Germany) and labelled angle α. The medial wall of the acetabulum was also outlined and labelled Line B. The angle between Line B and the line intersecting the axial plane and the sagittal plane was labelled angle β and measured using an MB ruler (Figure 1). The line that ran through the anterior and posterior points of the lateral brim of the acetabulum on the selected axial CT images was labelled Line C. The angle between Lines A and C was marked and measured, and was labelled angle γ (Figure 2).Sixteen pelvic specimens, which were harvested from the aforementioned sixteen cadaveric specimens, were selected for posterior column retrograde lag screw insertion. All of the specimens were stripped of soft tissue. They were put into a radiolucent prefabricated box and placed in the supine position on an operating table. Fluoroscopic guidance alone was used for screw insertion. A C-arm unit (Siemens Medical, Munchen, Germany) was used to establish the tangential views of both the PCPC and medial wall of the acetabulum of 16 bony pelvic specimens by referencing the unique angles α and β measured on the CT images for each specimen. The C arm was positioned according to the measured angles (Figure 3). The angular marking on the C arm was used to confirm the angles for the image intensifier. The intra-operative correction of the C-arm position was not performed. A lag screw was inserted in retrograde fashion into the posterior column as close to the posterior cortex as possible under the fluoroscopic guidance of the iliac oblique view and two tangential views. On the iliac oblique view, the lag screw was placed medial to the subchondral bone of the acetabulum. On the tangential view of the PCPC, the lag screw was placed medial and adjacent to the PCPC. On the tangential view of the medial wall of the acetabulum, the lag screw was inserted lateral to the medial wall. CT scans were then obtained to document the position of the lag screws.

To confirm that the angles α, β and γ measured in human volunteers are similar to those observed in the cadaveric specimens, 138 volunteers who were scheduled to undergo CT scanning of the pelvis for suspected avascular necrosis of the femoral head were screened. Eighty-six volunteers who were subsequently found to have evidence of femoral head pathology, bony deformity of the pelvis, or evidence of prior acetabular trauma or surgery were excluded. The remaining 52 volunteers were enrolled in this study and provided informed consent to participate in the study. Neither monetary nor non-monetary compensation was provided to these subjects. There were 27 males and 25 females, with a mean age of 48 years (range, 31–69 years), mean height of 172 cm (range, 163–186 cm) and mean weight of 68.2 kg (range, 56–89 kg). A lead garment was used to protect the volunteers from unnecessary radiation during CT scanning. Axial CT images of the volunteers were obtained according to the same parameters as for the cadaveric specimens. Angles α, β and γ were measured on the axial CT images following the same method as for the specimens. In the current study, the screw insertion itself was only performed on cadavers and not on the healthy volunteers. The Institutional Review Board of the Third Hospital of Hebei Medical University approved this study after thorough examination and verification.

### Statistical analysis

Statistical analyses were performed using SPSS 13.0 for Windows (SPSS, Chicago, IL, USA). Values were expressed as the mean ± standard deviation (SD). The two-tailed *t* test was applied to analyse these variables. A *p* value of <0.05 was considered significant.

## Results

**Figure 4:**
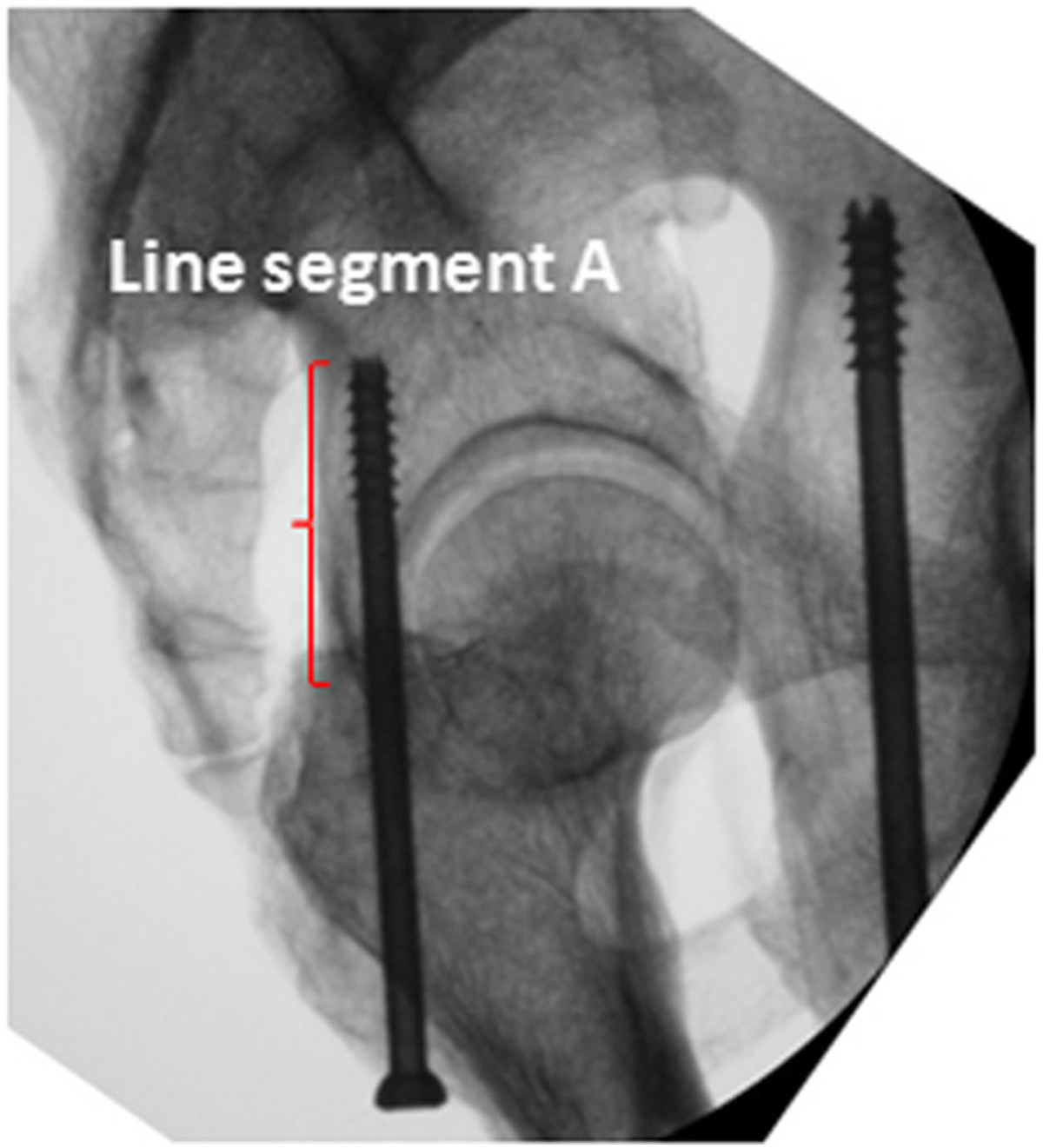
**On the tangential view of the posterior cortex of the posterior column, the posterior cortex appears as a nearly straight-line segment between the lesser and greater sciatic notches (Line segment A, the red curly brace).**

**Figure 5:**
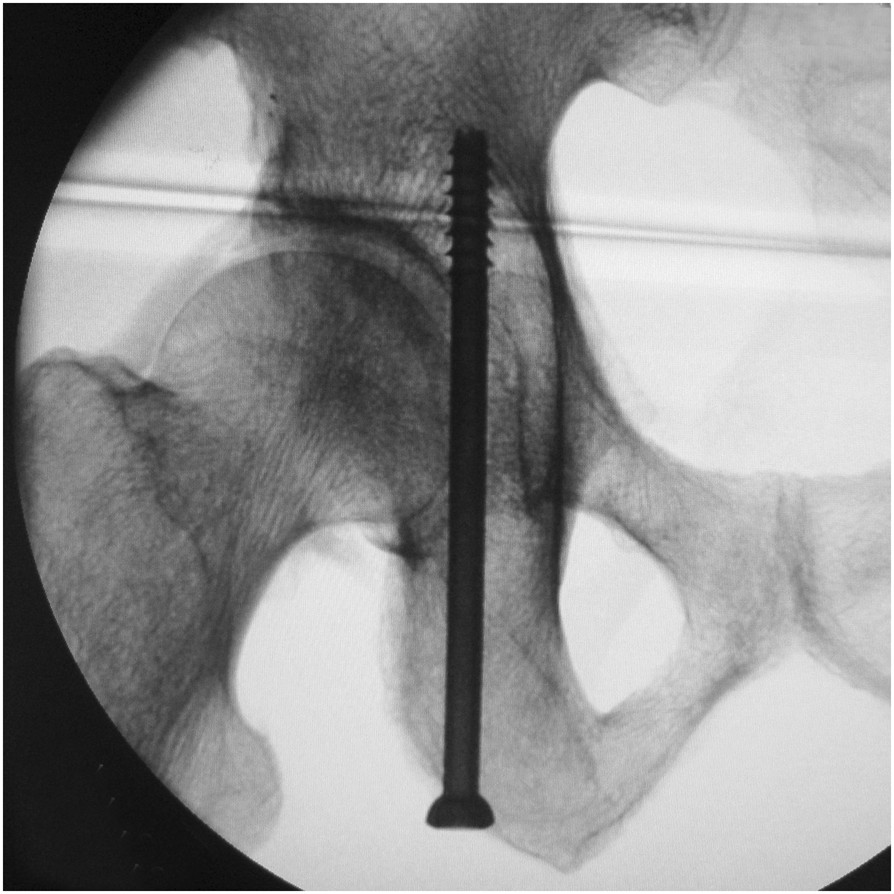
**On the tangential view of the medial wall of the acetabulum, the medial wall appears as a distinct straight line.**

**Figure 6:**
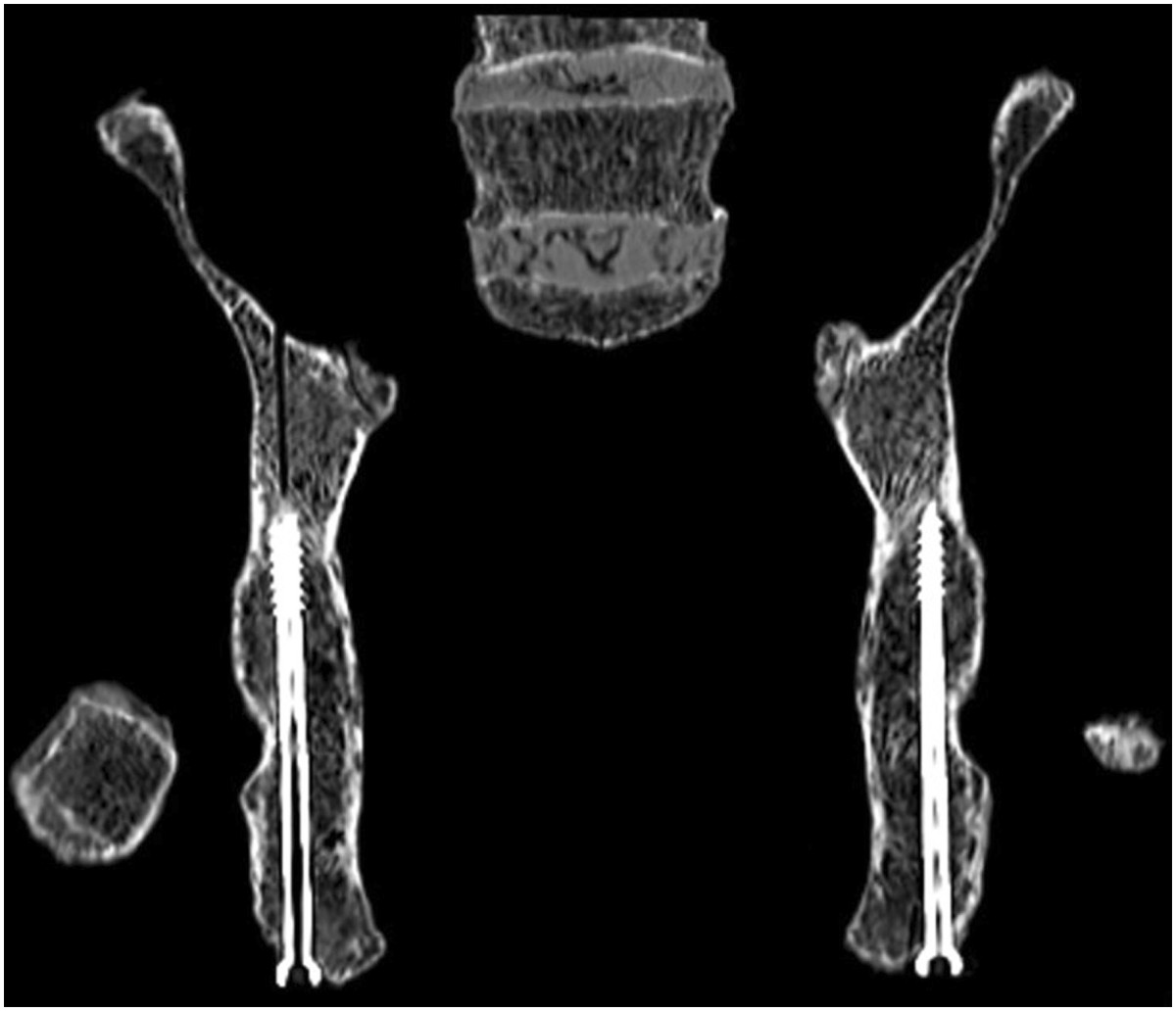
**The oblique coronal reconstructed CT images shows that the full length of the lag screw is in the bony corridor of the posterior column, and the shortest distance between the posterior cortex and lag screw was at the levels of the greater and lesser sciatic notches.**

The angles α, β and γ measured on the CT images at the level of the middle height of the acetabulum of specimens are summarised in Table 1. The mean values of angles α, β and γ for specimens were 29.3 (range, 26.5-34.2), 8.1 (range, 6.9-9.8) and 93.7 (range, 85.7-103.6) degrees, respectively. While collecting the obturator oblique radiographs of the acetabulum, the direction of the radiological beam was at an angle of 45 degrees with the line intersecting the axial plane and the coronal plane. Line A was at an angle of approximately 30 degrees with the line intersecting the axial plane and the coronal plane. Accordingly, Line A was at an angle of approximately 15 degrees with the direction of the radiological beam when collecting the obturator oblique view. That is to say, the tangential projection of the PCPC can be obtained by rotating the C-arm fluoroscopic beam 15 degrees outward from the position where the obturator oblique view is collected. On this view, the projection of the PCPC appears as a nearly straight line segment (Line segment A) between the lesser and greater sciatic notches (Figure 4). Line C was nearly perpendicular to line A, which means that the entire outline of the acetabulum can be almost demonstrated on the tangential views of the PCPC. In a similar way, the tangential view of the medial wall of the acetabulum can be obtained by rotating the radiological beam angle β outward from the position where the anteroposterior view of pelvis is taken. On this view, the medial wall also appears as a distinct straight line (Figure 5).The lag screws were safely inserted into the posterior columns of the pelvic specimens under fluoroscopic control in the iliac oblique view and the tangential views of both the PCPC and medial wall of the acetabulum. During the procedures, there were no failures in inserting the screws into the proper position. The CT images of the specimens confirmed that the lag screws remained within the osseous corridor of the posterior column throughout. The shortest distance between the posterior cortex and lag screw was at the level of the greater and lesser sciatic notches, which was demonstrated on the oblique coronal reconstructed CT images (Figure 6). On the tangential views of the PCPC, the bony cortices were closest to the intraosseous lag screws at the levels of the greater and lesser sciatic notches, which was apparent in the oblique coronal CT images (Figure 4).

**Table 1:** **The angles** α**,** β **and** γ **measured on the selected axial CT images for both specimens and volunteers**

Angles	Specimens ($\bar{\chi }\pm s$) (range	Volunteers ($\bar{\chi }\pm s$) (range)	
	(n = 16)	Male (n = 27)	Female (n = 25)
***Angel*** α	29.3 ± 2.8 (26.5-34.2)	30.4 ± 4.1 (25.9-36.5)	28.5 ± 3.7 (24.5-33.8)
***Angle*** β	8.1 ± 1.4 (6.9-9.8)	9.2 ± 1.9 (6.3-11.9)	7.7 ± 1.8* (5.2-10.3)
***Angel*** γ	93.7 ± 5.6 (85.7-103.6)	91.6 ± 4.8 (85.2-101.4)	93.9 ± 5.2 (86.9-103.7)

*There was statistically significant difference on angle β between male and female volunteers.

The angles α, β and γ measured on the CT images at the level of the middle height of the acetabulum of volunteers are summarised in Table 1. The angles α, β and γ were 30.4 ± 4.1 (range, 25.9-36.5), 9.2 ± 1.9 (range, 6.3-11.9) and 91.6 ± 4.8 (range, 85.2-101.4) degrees for male volunteers and 28.5 ± 3.7 (range, 24.5-33.8), 7.7 ± 1.8 (range, 5.2-10.3) and 93.9 ± 5.2 (range, 86.9-103.7) degrees for female volunteers, respectively. No statistically significant differences were found between the specimens and male volunteers for angles α, β or γ (*P* = 0.359, *P* = 0.067, *P* = 0.210, respectively). For volunteers, there were no statistically significant differences between male and female volunteers for angles α and γ (*P* = 0.092, *P* = 0.111, respectively); however, angle β measured on males was significantly larger than that on females (*P* = 0.006).

## Discussion

In the current study, the axial CT images at the middle height level of the acetabulum were obtained for both cadaveric specimens and volunteers. The angles between the PCPC and the line intersecting the axial plane and the coronal plane and those between the medial wall of the acetabulum and the line intersecting the axial plane and the sagittal plane were measured. The radiological beams during collection of the obturator oblique views of the acetabulum were at an angle of approximately 15 degrees with the PCPC. Namely, the tangential view of the PCPC can be taken by rotating the C-arm unit 15 degrees outward from the position where the obturator oblique view is taken. The tangential views of the medial wall of the acetabulum can be obtained by rotating the C-arm unit outward approximately 9.2 (for males) or 7.7 degrees (for females) from the position where the anteroposterior view of pelvis is taken. In all cases, lag screws inserted into the posterior column, under the guidance of the oblique iliac view and the tangential views of both the PCPC and medial wall of the acetabulum, remained clear of the hip joint and adjacent neurovascular structures and intrapelvic organs.

Intraoperative and postoperative radiographic imaging has been commonly used to evaluate possible joint penetration by periacetabular screws[24–28]. Postoperative CT is generally accepted as the most accurate technique for the detection of intra-articular screws[25–27]. However, intraoperative CT scans are not available in the majority of operating rooms, especially in developing countries. Fortunately, intraoperative fluoroscopy has been recently considered to have similar accuracy when compared to postoperative CT scans in detecting the misplacement of periacetabular screws[28]. Another obvious advantage of fluoroscopy is that data obtained in the operating room can be used immediately, and corrective action can be taken before the completion of the surgical procedure, theoretically reducing the need for reoperation[15]. Fluoroscopic navigation is a relatively new technique with numerous potential applications in the field of orthopaedic trauma[29]. Recently, 2-dimensional and 3-dimensional fluoroscopic navigation procedures were introduced for acetabular fracture surgery. Both navigation procedures can increase the precision of screw placement, prevent intraarticular penetration during drilling, obviate the need for repeated imaging in multiple planes and decrease radiation exposure for both the patients and surgeons[30, 31]. However, fluoroscopic navigation requires specialised equipment and instruments, and it is not available everywhere[29]. Therefore, techniques that rely on standard intra-operative fluoroscopy alone may be beneficial in facilitating the safe insertion of percutaneous posterior column lag screw.

Percutaneous screw fixation of a posterior column fracture has been a challenging task because of its unique and complex anatomy as well as the risk of penetration of the hip joint, damaging the adjacent neurovascular bundles or intrapelvic organs. Therefore, a technique for precise insertion of percutaneous screws requires knowledge of the 3-dimensional anatomy of the acetabulum and guidance with intraoperative fluoroscopy. Radiological evaluation, including an anteroposterior view, iliac oblique and obturator oblique views[16–22], is employed during retrograde fixation of the posterior column using a lag screw. The iliac oblique view can be taken as a good reference for avoiding penetration of the hip joint[1]. However, there are still no specific views for demonstrating the PCPC and the medial wall of the acetabulum, which can be used to avoid damaging the neurovascular structures and intrapelvic organs by lag screws.

In the current study, we introduced the tangential views of the PCPC and the medial wall of the acetabulum. The tangential views of the PCPC can be obtained using the C-arm fluoroscopic unit during the operation. The PCPC overlapped as a nearly straight-line segment between the lesser and greater sciatic notches as shown in Figure 5. From this view, the lag screw inserted medial to the straight line can ensure that the posterior cortex will not be protruded and that the neurovascular bundles, including the inferior gluteal nerve, inferior gluteal arteries and sciatic nerve, will not be injured. The lag screw adjacent to Line segment A can also guarantee that the screw is away from the hip joint and will not lead to intra-articular penetration. The tangential view of the medial wall of the acetabulum can be used to ensure that the lag screw is placed lateral to the medial wall and leaves the intrapelvic organs uninjured. Previous anatomical study has demonstrated that the thinnest part of posterior column is at the level of the middle height of the acetabulum[23]. Therefore, appropriate positioning and directionality of lag screw at this level, as illustrated on the two tangential views and iliac oblique views, can help to estimate the pathway of the full length of the screw. Following the three views, we inserted the lag screws into the posterior columns of 16 bony pelvic specimens, and subsequent CT scans confirmed that the posterior columns safely accommodated the screws.

The limitations of this study include the small sample size of the specimens and volunteers. The angles obtained in the study only represent the radiological features of a fraction of adult populations. Another limitation is that we have not confirmed the effectiveness of this technique in ensuring appropriate screw placement in patients with posterior column fractures. We plan to use these tangential views in the clinical setting to further confirm the technique’s effectiveness and validity in safe fixation of posterior column fractures using lag screws.

## Conclusions

Lag screws can be safely inserted into the posterior column under the guidance of the oblique iliac view and the tangential views of the PCPC and medial wall of the acetabulum. The angles between the PCPC and the line intersecting the axial plane and the coronal plane and those between the medial wall of the acetabulum and the line intersecting the axial plane and the sagittal plane were measured on the axial CT images at the middle height level of the acetabulum. The tangential view of the PCPC can be taken by rotating the C-arm unit approximately 15 degrees outward from the position where the obturator oblique view is taken. The tangential views of the medial wall of the acetabulum can be obtained by rotating the C-arm unit outward approximately 9.2 (for males) or 7.7 degrees (for females) from the position where the anteroposterior view of pelvis is taken.

## Authors’ original submitted files for images

Below are the links to the authors’ original submitted files for images. Authors’ original file for figure 1 Authors’ original file for figure 2 Authors’ original file for figure 3 Authors’ original file for figure 4 Authors’ original file for figure 5 Authors’ original file for figure 6

## Abbreviations

CT: Computed tomography
PCPC: Posterior cortex of the posterior column.
