# Supplementary figures and images for: Fluoroscopic views for safe insertion of lag screws into the posterior column of the acetabulum
Source: BMC Musculoskelet Disord. 2014 Sep 15;15:303. doi: 10.1186/1471-2474-15-303 (PMC4169822; doi:10.1186/1471-2474-15-303)

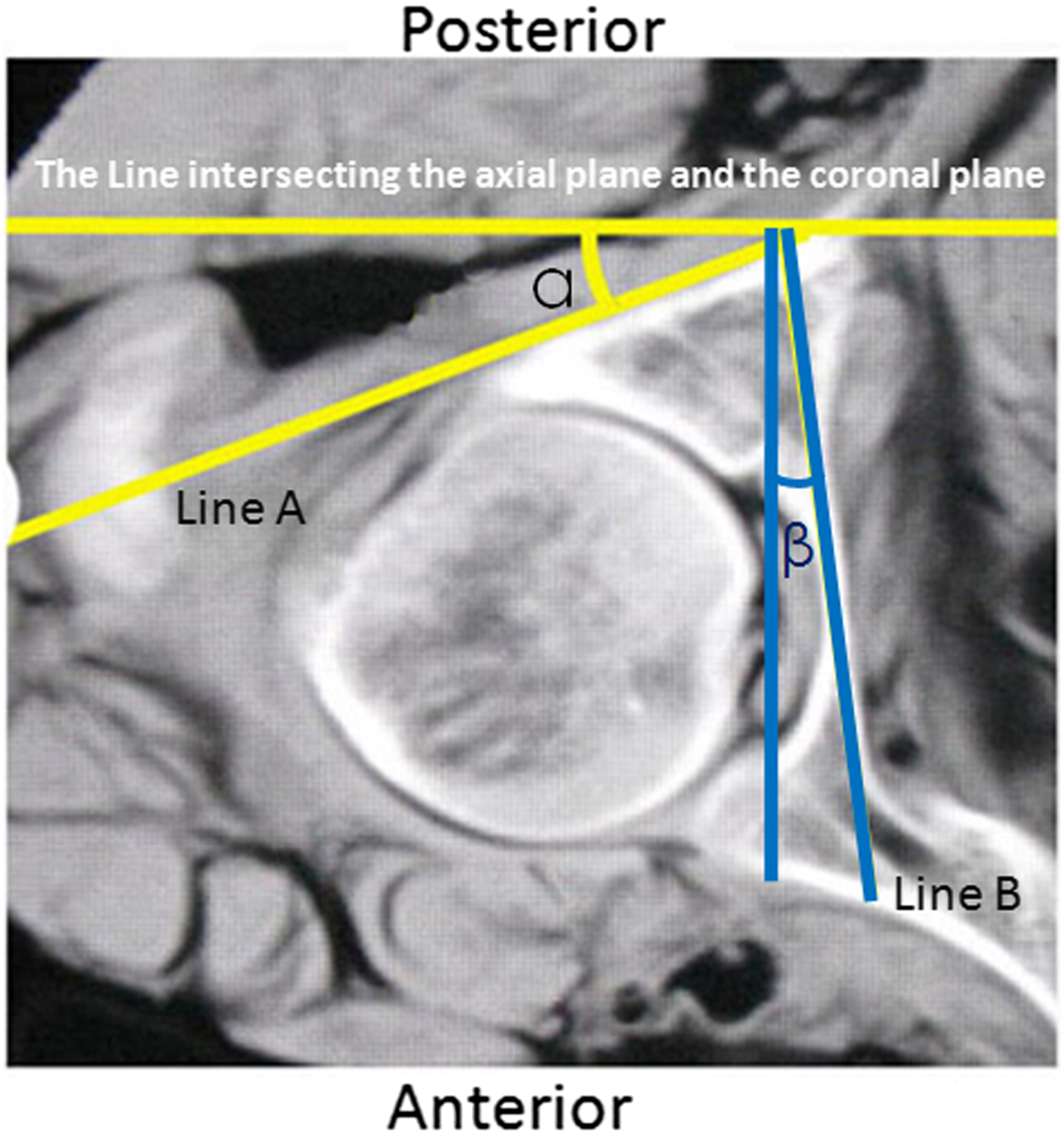

Supplement: Supplementary file 1 — Authors’ original file for figure 1 [file 12891_2014_2243_MOESM1_ESM.tif]

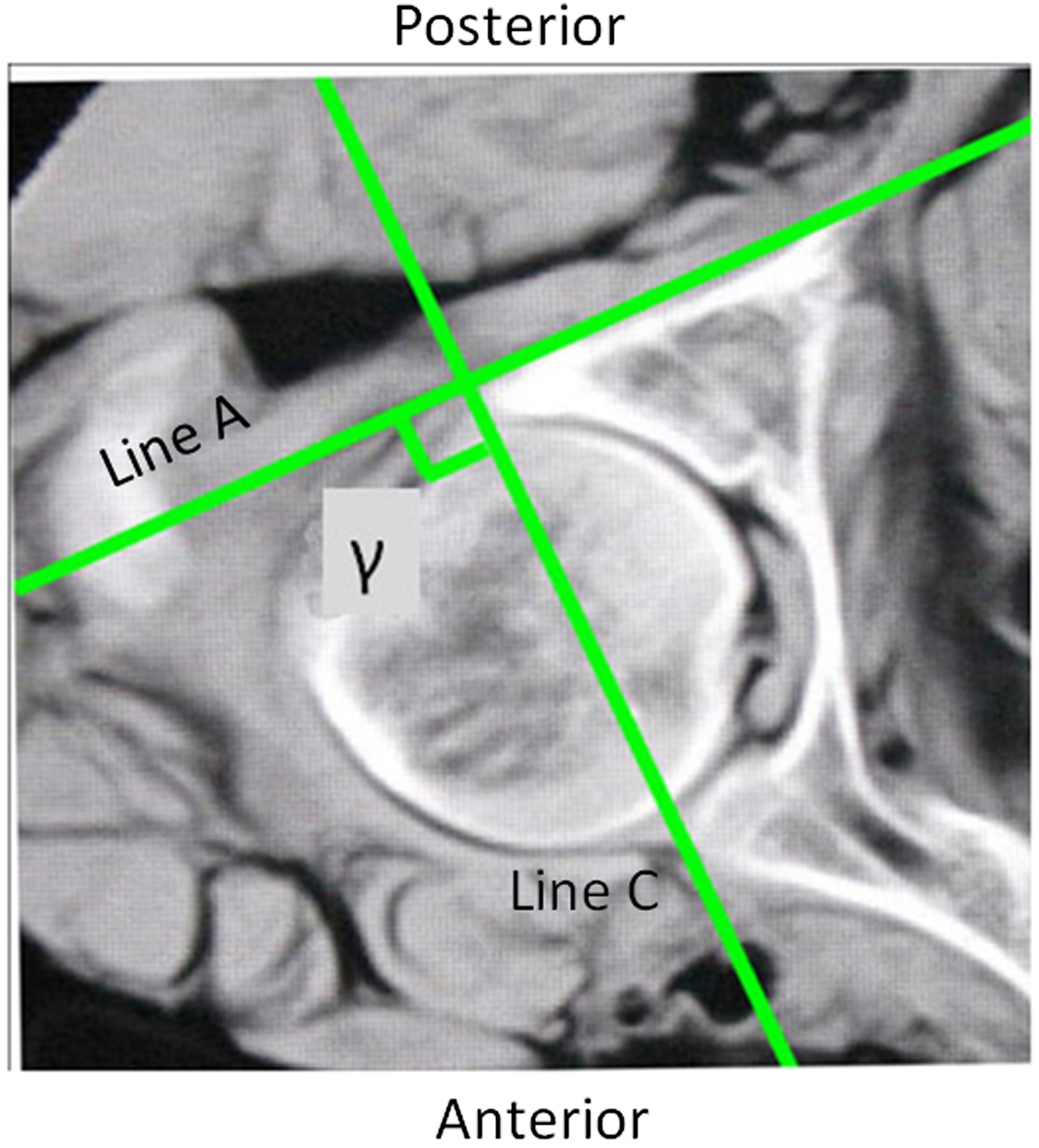

Supplement: Supplementary file 2 — Authors’ original file for figure 2 [file 12891_2014_2243_MOESM2_ESM.tif]

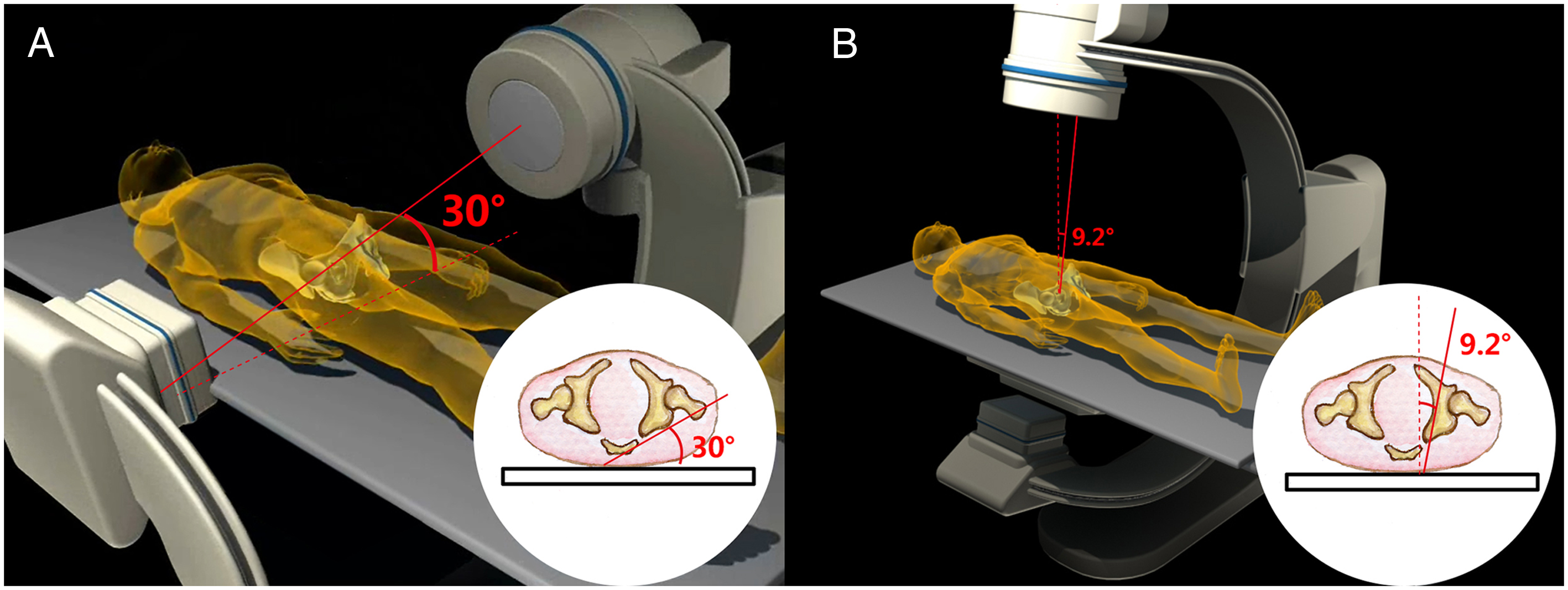

Supplement: Supplementary file 3 — Authors’ original file for figure 3 [file 12891_2014_2243_MOESM3_ESM.tif]

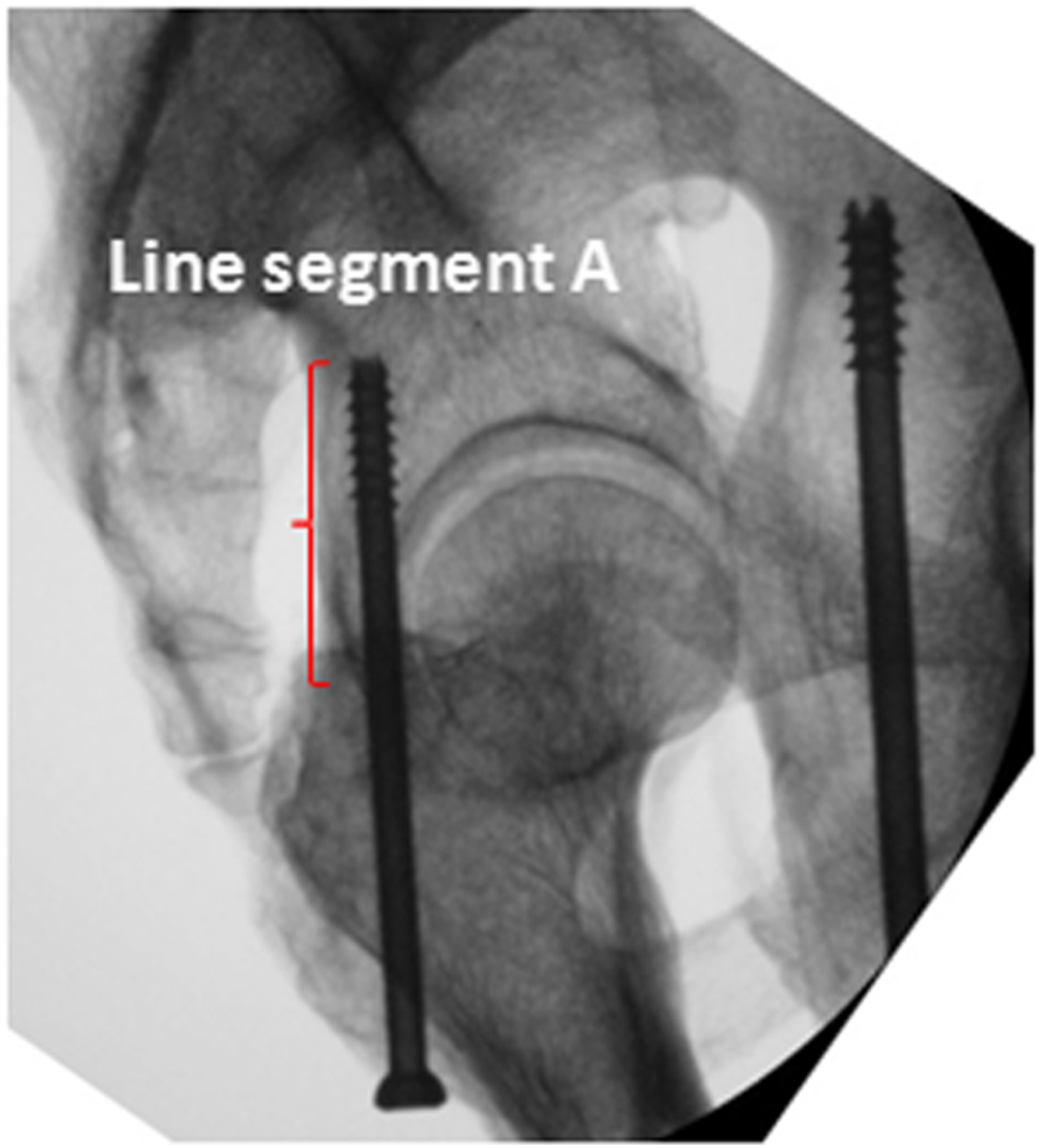

Supplement: Supplementary file 4 — Authors’ original file for figure 4 [file 12891_2014_2243_MOESM4_ESM.tif]

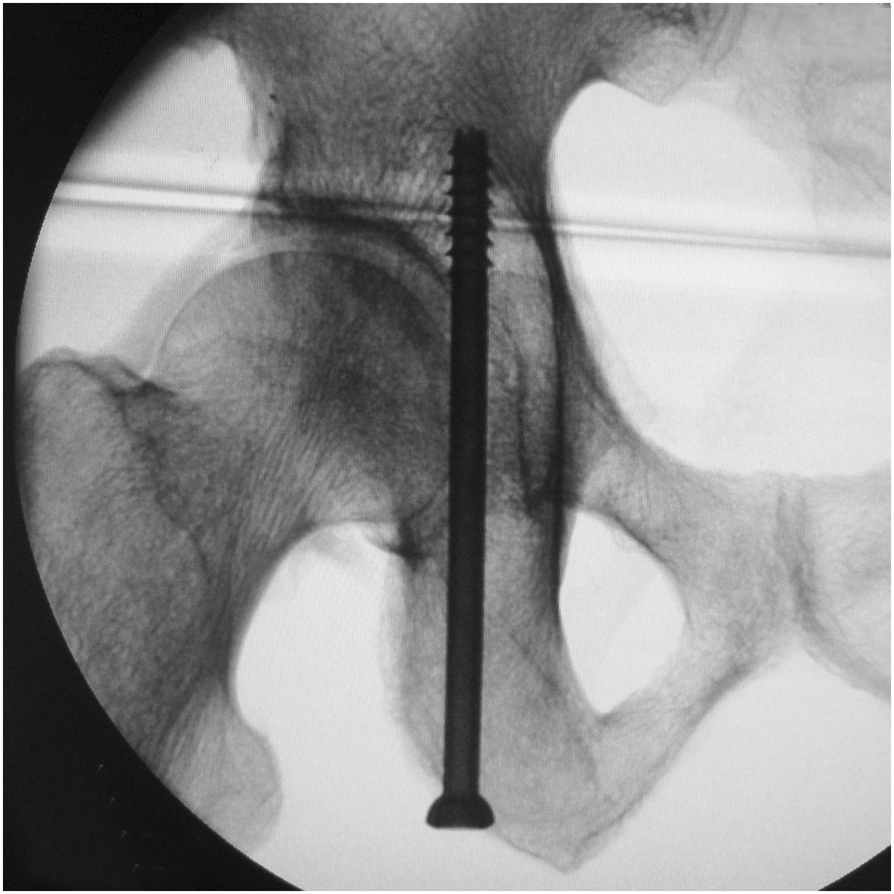

Supplement: Supplementary file 5 — Authors’ original file for figure 5 [file 12891_2014_2243_MOESM5_ESM.tif]

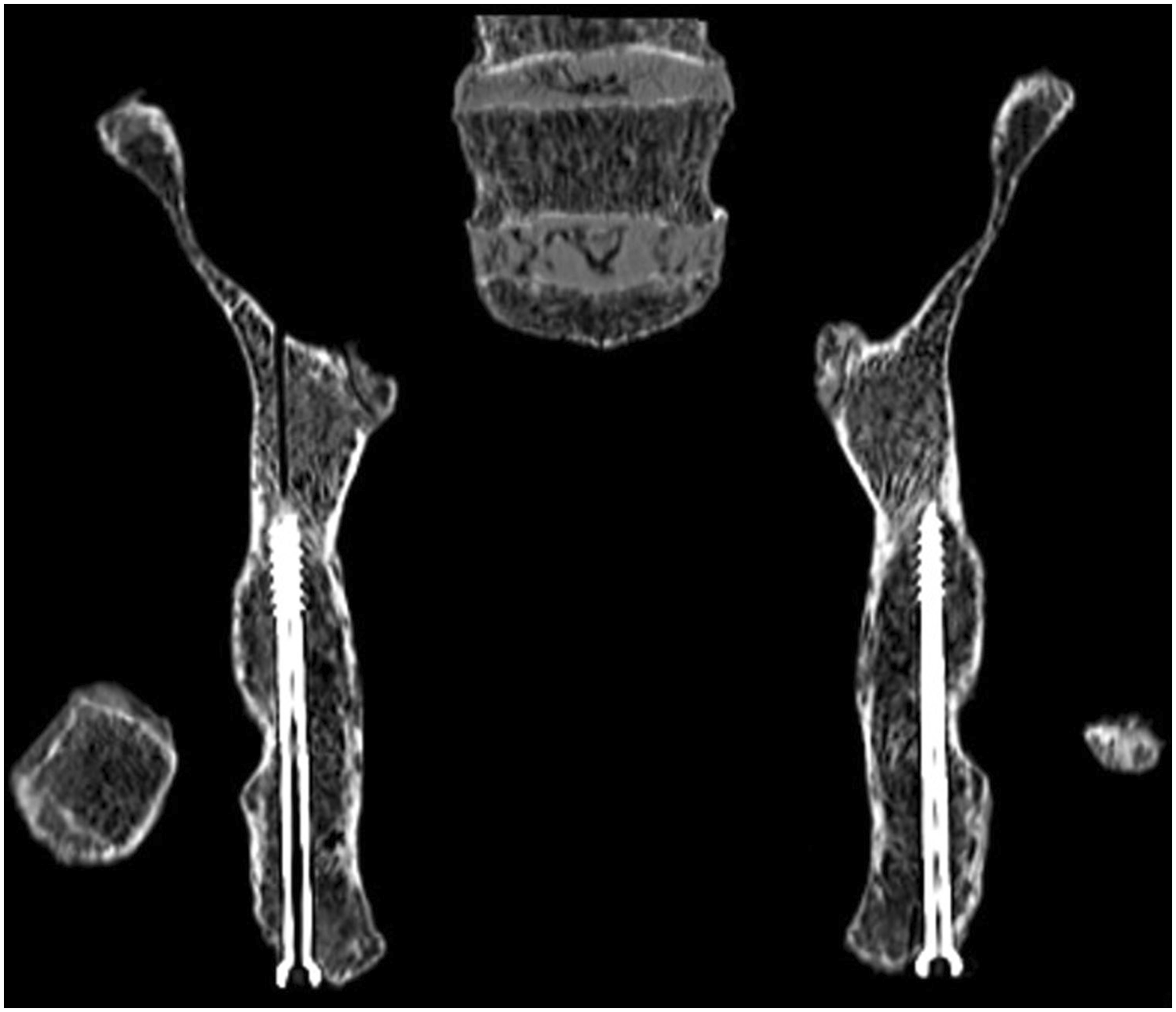

Supplement: Supplementary file 6 — Authors’ original file for figure 6 [file 12891_2014_2243_MOESM6_ESM.tif]
